# Does the Presence of the Cilioretinal Artery Affect the Incidence, Clinical Picture and Progression of Age-Related Macular Degeneration?

**DOI:** 10.3390/diagnostics13091593

**Published:** 2023-04-29

**Authors:** Elżbieta Krytkowska, Aleksandra Grabowicz, Krzysztof Safranow, Anna Machalińska

**Affiliations:** 1 First Department of Ophthalmology, Pomeranian Medical University, 70-111 Szczecin, Poland; 2 Department of Biochemistry and Medical Chemistry, Pomeranian Medical University, 70-111 Szczecin, Poland

**Keywords:** cilioretinal artery, age-related macular degeneration (AMD), choroidal thickness, CFH Y403H, ARMS2 A69S

## Abstract

The aims of this study were to analyze the relationship between the presence of the cilioretinal artery (CRA) and the incidence, severity and progression of age-related macular degeneration (AMD) and to estimate the influence of the CRA on choroidal and retinal parameters. A total of 287 patients with AMD and 110 healthy controls were enrolled in the study. CRA occurrence was determined using color fundus images. AMD progression was assessed after 3 years. There was no difference in the incidence of CRA between the AMD and control groups (23.34% vs. 24.55%; *p* = 0.8). Lower-stage AMD was more frequently observed in eyes with the CRA than in eyes without the artery (*p* = 0.016). The CRA did not influence disease progression (*p* = 0.79). The CRA did not influence retinal and choroidal thickness and volume parameters or the retinal vessel caliber and functionality in either the AMD or control groups. There was no relationship between CRA presence and CFH Y402H and ARMS2 A69S risk variants. The results did not show a protective effect of the CRA on the incidence and progression of AMD. The CRA may affect the severity of AMD; however, the mechanism of this phenomenon is unclear.

## 1. Introduction

Age-related macular degeneration (AMD) is one of the greatest challenges of ophthalmology. It is a chronic, progressive disease of the central retina that occurs mainly in people over 50 years of age, affecting approximately 2.5% of the world’s population [1]. AMD is a disease with a complex pathomechanism that has not been fully explained, in which damage occurs to the photoreceptors and their support complex consisting of the retinal pigment epithelium (RPE), Bruch’s membrane (BrM) and choriocapillaris [2]. Some researchers have indicated that the primary defect in AMD is impaired blood flow in the choroid [3,4]. The results of numerous histopathological and imaging studies show damage to the choriocapillaris and reduced blood flow in all layers of the choroid in eyes with AMD [4,5,6,7]. These disturbances increase with the duration of the disease and its advancement. Retinal microcirculation status may reflect generalized disturbances in cardiovascular diseases, while risk factors for systemic vascular disease also increase the risk of developing macular degeneration.

The cilioretinal artery (CRA) is the most common congenital variant of the retinal circulation. It arises directly from the posterior ciliary artery or from the choroid, providing additional blood supply to the retina [8]. This is observed in eyes with central retinal artery occlusion, when preserved flow in the CRA is associated with better visual acuity and maintenance of the central visual field. It has been documented that CRA may provide additional blood supply to the temporal part of the optic disc, which is mainly supplied by the posterior ciliary artery, and thus may affect the preservation of the visual field in patients with glaucoma [9,10]. In 1994, Winther-Tham and Lindblom hypothesized that the presence of the cilioretinal artery may have a protective effect on the development of wet AMD by providing an additional source of blood for the hypoxic retina [11]. The analyses of the protective role of the CRA on AMD development were then further replicated in a larger group of AMD patients, but conflicting results were obtained [12,13]. To the best of our knowledge, the relationship between the presence of the CRA and choroidal parameters in eyes with AMD has not been studied thus far. Previously, we reported that CFH Y402H and ARMS2 A69S polymorphisms may have an impact on retinal microcirculation [14]. However, an analysis of the presence of high-risk AMD variants in the context of CRA prevalence has not yet been performed.

Thus, the aim of the current study was to investigate whether the presence of the CRA was an important vascular factor in the pathogenesis and progression of AMD. In addition, we wanted to analyze the effect of the presence of the CRA on retinal and choroidal morphology and function. Finally, we also investigated whether the presence of the CRA was associated with high-risk AMD polymorphisms.

## 2. Materials and Methods

### 2.1. Study Design

In this prospective study, patients with a diagnosis of AMD were enrolled, and patients without any features of macular degeneration were assigned to the control group. An informed consent form was signed by all patients before enrolment in the study. A detailed medical history, physical examination, ophthalmologic examination and genotyping for specific high-risk AMD variants were performed in the First Department of Ophthalmology of Pomeranian Medical University in Szczecin. After 36 months, eyes without late AMD at baseline were ophthalmologically re-examined for evidence of AMD progression and further analysed to determine the relationship between CRA presence and disease course.

### 2.2. Ophthalmologic Examination

All participants underwent comprehensive ophthalmologic examination, including best-corrected distance visual acuity (BCDVA) with logMAR chart, slit lamp biomicroscopy, and detailed fundus examination after pupil mydriasis with 1% tropicamide solution. Color fundus photographs were taken using a Topcon Non-mydriatic Retinal camera (Topcon Corporation, Tokyo, Japan). Macular scanning was performed using OCT Spectralis (Heidelberg Engineering, Heidelberg, Germany) with enhanced depth imaging (EDI) mode. For precise and reliable drusen subtype differentiation, fundus autofluorescence (FAF) and near infrared imaging (NIR) were performed. In order to minimize the influence of factors interfering with the measurements of choroidal parameters, Goldmann applanation tonometry and axial length calculation were performed. To confirm the presence of the macular neovascular membrane in doubtful cases, fluorescence angiography (FA) and/or angio-OCT (OCTA) was performed.

Exclusion criteria included disorders that could potentially affect the choroidal or retinal vasculature such as glaucoma, high myopia (≥6 D), retinal vessel occlusion, retinopathy of any type, uveitis, history of serious ocular trauma and ocular tumor. Only eyes with an axial length of 22–25 mm were further analysed. Imaging was performed exclusively in eyes with transparent optical media to obtain the highest quality fundus photographs and OCT scans. A history of cataract surgery within the last 3 months or any prior laser therapy or vitreoretinal surgery were exclusion factors. Only anti-vascular endothelial growth factor (AVEGF) treatment-naïve AMD patients were initially recruited to the study.

### 2.3. CRA Identification

**Figure 1:**
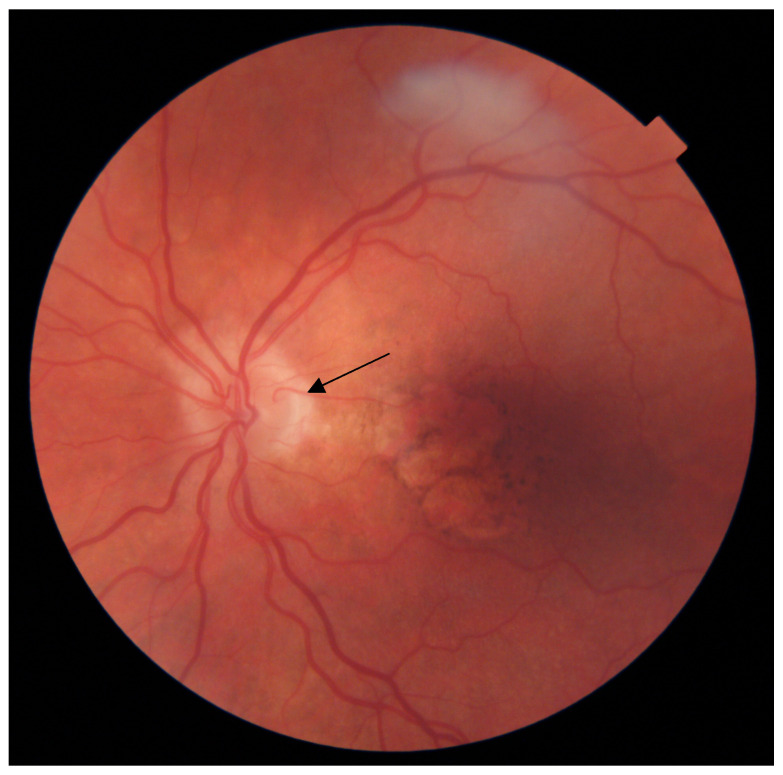
Cilioretinal artery (black arrow) in the eye of a 78-year-old woman with geographic atrophy secondary to confluent soft drusen resolution.

The presence of the cilioretinal artery was confirmed by a qualified ophthalmologist based on color fundus photographs. The cilioretinal artery was defined as a hook-like bending retinal artery without any apparent connection with the central retinal artery, originating from the margin of the optic disc and extending towards the retina. Only eyes in which the CRA started in the temporal region of the optic nerve head, ran towards the macula and reached the central area within 500 µm of the foveal center were analysed. Eyes with vessels with CRA morphology but originating from the nasal side of the optic disc and facing the nasal retina were classified as having no CRA. Similarly, very thin vessels (diameter <10% of central vascular structure) were excluded (Figure 1).

### 2.4. Optical Coherence Tomography

Enhanced-depth imaging was performed on both eyes from each subject using the Heidelberg Spectralis SD OCT (870 nm) device (Heidelberg Engineering, Heidelberg, Germany). The device performs 70,000 A scans per second, with an axial and transverse tissue resolution of 3.9 μm and 6 μm, respectively. All participants underwent macular OCT at the same time of the day, after 30 min of rest. The patients were instructed not to smoke for 6 h or drink any fluids for 1 h before the examination. The OCT imaging was performed after pupil dilation with 1% tropicamide solution. A macular 25° × 25° volume acquisition protocol was used to obtain 61 cross-sectional B-scans. To minimize image artifacts, an active vision tracking was used. According to the manufacturer guidelines, only images with a signal strength (Q score) greater than 25 dB were further analysed. The central retinal thickness was assessed in the central region of the ETDRS map using the inbuilt Spectralis software Heidelberg Eye Explorer (version 6.0c, Heidelberg Engineering, Heidelberg, Germany). CRT was defined as the vertical distance between the vitreoretinal surface and Bruch’s membrane.

Choroidal delineation was performed manually after the automated retinal layer segmentation software was disabled. Next, the reference lines of the built-in automated segmentation were moved from the retinal to the choroidal boundaries. This method enabled the use of built-in functions for automatic mapping of retinal thickness and volume. The details of the OCT image analysis process have been described previously [15].

For a more precise assessment of choroidal vasculature, we also calculated the choroidal vascularity index (CVI), using the semiautomated method described previously by Sonoda et al. with later modifications by Agrawal et al. [16,17]. An EDI OCT scan passing through the sub-foveal area was selected for further analysis. The choroidal area was binarized using ImageJ software (version 1.53e; Java 1.8.0_172, National Institutes of Health, Bethesda, MD, USA). Firstly, the region of choroid was marked on the B OCT scan using polygon tools. The upper border of the choroid was manually traced along the retinal pigment epithelium, while the lower border was marked along the choroid–scleral junction (CSI). After converting to 8-bit images, Niblac’s auto local threshold was applied. Brightness was reduced for better exposure of blood vessels and to minimize noise. The image was then converted to the red, green, blue (RGB) image. Next, the luminal area was determined using the color threshold tool. Three different thresholding steps (auto-threshold, Niblack’s auto local threshold and color threshold) were performed using the default settings in the ImageJ plugin. The choroidal vascularity index determined the ratio of the luminal area to the total choroidal area. Each measurement was repeated three times and averaged. Low-quality OCT scans, in which the course of the uveoscleral junction could not be unambiguously determined, were excluded from the study.

OCTA images were acquired in a 3 × 3 mm area using the Spectralis OCT Angiography Module (Heidelberg Engineering, Heidelberg, Germany). The Heidelberg TruTrack Active Eye Tracking system was used to reduce signal-to-noise ratio and motion artefact. Three-dimensional OCTA images and cross-sectional OCT images were co-registered, with automatic segmentation of the retinal and choroidal layers. The borders of the automated outlined slabs were checked for the propriate localization and corrected if needed. The presence of neovascular membrane was diagnosed by the presence of an abnormal flow signal in the images of the choroid and/or outer retina.

### 2.5. Phenotypic AMD Description

Dry AMD was diagnosed in eyes with drusen and/or pigmentary abnormalities, and/or geographic atrophy (GA) features were found via color fundus images and OCT. Active wet AMD was defined by the presence of the macular neovascular membrane, as confirmed by the presence of intra- and/or subretinal hemorrhages, exudations and/or vascularized PED on color fundus images, OCT scans or angiography, while inactive choroidal neovascularization (CNV) was diagnosed when macular scarring without the active features was present. Eyes with the presence of double layer sign (DLS) were assessed in detail for the presence of CNV, and fluorescein angiography and/or OCTA were performed in questionable cases.

AMD staging was based on changes observed in ophthalmoscopic examination and OCT images according to the Ferris system [18]. The retinologist assigned patients to one of three groups, depending on the severity of AMD. Group 1 (early AMD) included patients with medium drusen (63–125 μm) but without pigmentary. To group 2 (intermediate AMD) were assigned those patients who had large drusen or pigmentary abnormalities associated with at least medium drusen. Group 3 (late AMD) included patients with either advanced geographic atrophy or macular neovascularization of any type.

The type of drusen was classified based on its appearance in color fundus photography, OCT, NIR and FAF images. Hard drusen were punctate, small- to medium sized (<125 μm) lesions, yellow–white in color, with discrete borders, as observed in CFP, OCT and NIR images. Soft drusen were identified when yellow–white mound-like nodules with indistinct boundaries and gradually reduced density toward the periphery were observed in CFP images. On OCT scans, soft drusen were seen as moderately reflective dome-shaped sub-RPE deposits [19]. Pachy-drusen were identified on CFP as yellow–white deposits, isolated or scattered in the posterior pole, with better-defined and a more complex outer border compared to regular contour of soft drusen [20]. On OCT scans, pachy-drusen were often located near the pachy-vessels [21]. Subretinal drusenoid deposits (SDDs) were defined as the discrete white–yellowish deposits located in the perifoveal area. These corresponded with a round or triangular, well-defined, hyperreflective subretinal deposit accumulation of material that formed sharp peaks and may lead to abruption of the inner and outer photoreceptor segment layer on the OCT scans. On the FAF and IR images, the SDDs became more prominent and appeared as a hyper-auto-fluorescent/hyperreflective area in the center of the drusen surrounded by a hypo-reflective halo, creating the characteristic appearance of a target [22,23]. A diagnosis of pachy-choroid was established when the presence of thick choroid (sub-foveal choroidal thickness >350 μm or an extrafoveal focus that exceeded the foveal choroidal thickness by at least 50 μm) and the presence of pachy-vessels was detected on the EDI-OCT scans [24,25,26]. Pachy-vessels were recognized when the dilated outer choroidal vessels compressing the overlying choriocapillaris and Sattler’s layers were observed on cross-section EDI-OCT images [24,27]. We also assessed the relationship between the presence of the CRA and the size of soft drusen in the AMD group. The largest soft drusen in the circle area with a diameter of 6 mm centred in the fovea was selected for further evaluation. Drusen size was calculated as the product of their height (distance between Bruch’s membrane and RPE at the highest point of the drusen) and their base width along the BrM.

At the follow-up examination, AMD progression was recognized when a change in disease severity in at least one eye from early to intermediate AMD was present or when the features of late-stage disease were observed in eyes previously classified as early or intermediate stage. When none of the signs of a more advanced stage of disease were found, no AMD progression was established. Depending on the presence of features of progression, patients were assigned to one of two study subgroups.

### 2.6. Retinal Vessel Analysis

The Retinal Vessel Analyzer (RVA) (Imedos GmbH) is a tool for the assessment of retinal vessel diameter that analyses its brightness profile using video sequences obtained with a fundus camera. The exam was conducted in a dimly lit room after adequate pupil dilation with 1% tropicamide solution. The principle of 15 min of rest before the examination was maintained to stabilize hemodynamic parameters. Similarly, to exclude factors that could distort the test results, patients were advised to refrain from drinking coffee or smoking cigarettes for at least 5 h before the examination. During the static vessel analysis (SVA), 30-degree retinal photographs of each subject were taken with the FF450 plus fundus camera (Zeiss AG, Jena, Germany), as previously described [14,28]. The images were further analysed using VISUALIS and VesselMap software (IMEDOS Systems, Ltd., Jena, Germany). The following parameters were used for the evaluation: central retinal arteriolar equivalent (CRAE), which refers to the diameter of the central retinal artery; central retinal venular equivalent (CRVE), which refers to the diameter of the central retinal vein; and arteriovenous ratio (AVR), which indicates the CRAE/CRVE ratio. The width of the selected vessels was expressed in units of measurement (UM). After the baseline vessel diameter measurement for 50 s, the 3 cycles of 20 s flicker provocation for autoregulatory dilation and 80 s of steady illumination were performed, during which the vessel diameter returned to baseline. Responses from 3 cycles were averaged. Only one selected retinal artery and retinal vein was examined in each eye.

### 2.7. Genotyping

7.5 mL venous blood samples were collected in EDTA tubes. They were centrifuged at 2000 rpm, 4 °C for 10 min. Next, red blood cells were lysed with ammonium chloride-based lysing buffer (BD Biosciences, Franklin Lakes, NJ, USA). Then, nucleated cells were counted, and DNA isolation was performed with a total DNA isolation kit (Macherey-Nagel, Düren, Germany) according to the manufacturer’s protocol. AMD risk polymorphisms were genotyped, as previously described [29]. In CFH, rs1061170, encoding a Y402H interchange, was genotyped by restriction analysis with EagI, HhaI and Hsp92II enzymes. In ARMS2, LOC387715 rs10490924, encoding an A69S inter-change, was determined by direct DNA sequencing using an Applied Biosystems 3130 XL instrument for DNA sequencing (Thermo Fisher Scientific, Waltham, MA, USA). Molecular analysis was performed by a team of bioinformaticians in accordance with generally accepted standards.

### 2.8. Statistical Analysis

The Mann–Whitney U test was used to compare quantitative and rank variables between groups, as the distributions of most quantitative variables were significantly different from a normal distribution. Qualitative variables were compared between study groups with chi-squared or Fisher’s exact tests. The Wilcoxon signed rank test was used in a group of patients with unilateral CRA, comparing eyes with and without CRA. A *p*- value < 0.05 was considered statistically significant.

## 3. Results

### 3.1. Prevalence of the CRA in the AMD and Control Groups

We enrolled a total of 287 patients with AMD. As a control group, we enrolled 110 healthy subjects matched in terms of age (mean: 73.1 years in AMD and 72.9 years in control groups; *p* = 0.5). Female participants accounted for 62.78% in the AMD group and 74.55% in the control group (*p* = 0.03). We found no differences in CRA prevalence between the AMD and control groups (23.34% vs. 24.55%; *p* = 0.8). Bilateral distribution of the CRA was observed in 5.6% of AMD subjects and 3.9% of controls (*p* = 0.4). We did not find any correlation between CRA presence and age in either the AMD (*p* = 0.31) or control (*p* = 0.27) groups. Likewise, the gender distribution between CRA-positive and CRA-negative subjects was similar (*p* = 0.05 in the AMD group and *p* = 0.61 in the control group). The patients with and without CRA in both the AMD and control groups were not significantly different with regard to age and well-known atherosclerotic risk factors, including hypertension, history of ischemic heart disease, cardiac infarction, cerebral stroke, peripheral artery disease and smoking status. There were no significant differences in BMI, WHR, MAP or physical activity between the groups (Table 1).

### 3.2. The Impact of CRA on the Clinical Picture of AMD

Next, we aimed to assess the influence of CRA presence on disease characteristics in the AMD group. Importantly, there was a significant difference in AMD stage distribution, with a lower percentages of eyes with more advanced AMD in the group with CRA than in the group without CRA (*p* = 0.016). We found no association between CRA prevalence and soft drusen (*p* = 0.17) or their size (*p* = 0.29), AMD subtype (CNV and geographic atrophy: *p* = 1.0 and *p* = 0.22, respectively) or prevalence of pachy-choroid (*p* = 0.58), pachy-vessels (*p* = 0.61), pachy-drusen (*p* = 0.93) and SDD (*p* = 0.61) (Table 2).

### 3.3. Influence of the CRA on AMD Progression

Next, we aimed to assess the effects of CRA presence on AMD worsening. For this purpose, 94 patients with a diagnosis of early- or intermediate-stage AMD in at least one eye were followed up over 36 months. After 3 years, 18.6% of AMD participants with CRA and 21.43% without CRA presented with disease progression (*p* = 0.79); thus, we concluded that CRA presence had no impact on disease advancement.

### 3.4. Impact of the CRA on Clinical and Morphologic Retinal and Choroidal Parameters

We next analyzed whether clinical features, e.g., visual acuity and choroidal and retinal thickness and volume, were associated with the presence of CRA (Table 3). We found no differences in visual acuity between eyes with and without CRA in either the control or AMD groups (*p* = 0.3 and *p* = 0.72, respectively). We found that neither the choroidal thickness nor volume in the central region of the ETDRS ring differed between eyes with and without CRA in both the AMD (*p* = 0.14 and *p* = 0.14, respectively) and control (*p* = 0.99 and *p* = 0.99, respectively) groups. Accordingly, we found no differences in total choroidal volume in AMD (*p* = 0.09) and control (*p* = 0.63) eyes. Interestingly, in the control group, the CVI appeared to be higher in subjects with the CRA (*p* = 0.04), while no such relationship was observed in the AMD eyes (*p* = 0.92). There was no association between retinal thickness and CRA in either the control or AMD groups (*p* = 0.23 and *p* = 0.65, respectively).

Next, we considered the static and dynamic retinal vessel characteristics. There were no statistically significant differences in AVR between the presence and absence of CRA in either the control (*p* = 0.91) group or the AMD (*p* = 0.28) group. Similarly, the reactivity of retinal arteries and veins was not significantly different depending on CRA presence in either the control (*p* = 0.98, *p* = 0.93, respectively) or AMD (*p* = 0.87, *p* = 0.73, respectively) groups.

### 3.5. Associations between CFH Y402H and ARMS2 A69S Polymorphisms and CRA Prevalence

In the next step, we aimed to explore whether well-defined SNPs associated with an increased risk of AMD (CFH Y402H and ARMS2 A69S) were associated with CRA incidence in our patients. None of the genotypes in the two tested SNPs were associated with CRA presence in either the AMD or control groups (Table 4).

## 4. Discussion

The cilioretinal artery is the most common congenital anomaly of the retinal circulation [30]. It arises either directly from the choroid or from one of the posterior ciliary arteries, providing an additional or alternative blood supply to the retina [8]. The incidence of CRA varies with fundus imaging technique, with fluorescein angiography being the most sensitive for its detection. During AF, the CRA is observed to fill with dye at the same time as the choroidal circulation. However, in some cases, the filling of the CRA occurs simultaneously with the retinal circulation, which makes it impossible to identify by this method [31]. In studies based on fluorescein angiography, CRA prevalence was reported in 38.2–49.5% of cases, while studies using CFP showed that CRA occurred in 6.9–35% of healthy patients [31,32,33,34]. Bilateral incidence was estimated at 7–14.6% [31,32,34]. In our study, based on color and red-free fundus photography, a unilateral cilioretinal artery was visualized in 23.34% of AMD patients and 24.55% of controls, while bilateral distribution of the CRA was observed in 5.6% of AMD subjects and 3.9% of controls. The results of the current study are consistent with those of previous reports [12,13,32,35]. It is worth mentioning that cases of bilateral ciliary artery were less frequent in our study compared to those mentioned above. A possible explanation is that, in the current study, only cases of CRA located in the temporal part of the optic disc were analyzed.

Importantly, we found no differences in CRA prevalence between the AMD and control groups. This is consistent with Bavinger et al., who analyzed 350 patients from the CATT study and found that the presence of the retinal ciliary artery was not associated with a lower incidence of either CNV or GA [13]. On the other hand, in a study by Winther-Tham et al. from 1994, based on the analysis of 100 eyes with wet AMD, a lower incidence of exudative AMD in eyes with CRA compared to eyes without this artery was noted, indicating that its presence may compensate to some extent for the reduced nutrient supply to the macula [11]. Similar results were obtained 20 years later in a study by Snyder et al. based on a much larger group of 3600 patients from the Age-Related Eye Disease Studies (AREDS) [12]. The authors reported that the presence of the cilioretinal artery was associated with a lower CNV prevalence and lower AMD severity score. The same results were observed when a group of eyes with CRA were compared with eyes without CRA, as well as when a subgroup of AREDS participants with unilateral CRA was analyzed [12]. Similar to the Snyder group, we found that CRA-positive eyes with AMD had less severe disease than eyes without CRA. Similar results were reported by Kim et al. comparing CRA-positive and CRA-negative fellow eyes of AMD patients [36]. These results indicate that the presence of the CRA may influence the AMD course. However, our study results failed to show the influence of CRA presence on AMD progression at the 3-year follow-up. Snyder et al. previously reported a lower rate of progression to late-stage AMD, as well as a lower prevalence of CNV, in eyes with the CRA in a 5-year follow-up. However, they found no change in AMD severity score and no difference in developing GA compared to the non-CRA fellow eyes over 5 years of follow-up [12]. In contrast, in the study by Bevinger et al., over the same follow-up period, no association was found between the presence of the CRA and the development of choroidal neovascularization or geographic atrophy [13]. Although the results of the above studies are convergent in terms of the effect of the CRA on AMD progression, it should be noted that both studies were interventional. In the AREDS study, patients were supplemented with a set of antioxidants that was proven to significantly slow the progression of the disease, especially in eyes with unilaterally advanced disease. The CATT study evaluated the efficacy of intravitreal injections of AVEGF agents in patients with unilateral choroidal neovascularization. As this feature is a strong risk factor for AMD progression, it may have made the impact of the CRA on progression less apparent. Unlike the abovementioned studies, our study was purely observational. In addition, in the current study, the definition of progression included a change in disease status, and not only progression to the late stage of the disease. Moreover, none of the above studies included a control group of healthy eyes to better highlight possible differences related to the presence of the CRA.

Several theories have been formulated to explain the existing clinical data. It is possible that the CRA provides an additional significant source of blood for the central retina in AMD eyes. It was previously shown that, in acute retinal circulatory failure due to occlusion of the central retinal artery, eyes with preserved cilioretinal artery flow presented better visual acuity and perimetry scores than those without the CRA [37]. In AMD, the retinal photoreceptors are gradually damaged and atrophied. Growing dysfunction leads to increased degeneration of the retina, which, in a vicious cycle, leads to further dysfunction of cone and rod cells [38]. It is possible that the presence of an additional source of oxygen and nutrients from the CRA slows photoreceptor degeneration. However, we cannot exclude the possibility that the presence of the CRA in the macular area might have a negative impact on AMD, as the presence of a vessel directing blood from the choroid to the retina might adversely affect choroidal-dependent tissues, such as the macula. Shihab et al. previously reported the presence of more advanced glaucoma features in eyes with the CRA compared to eyes without the vessel [39]. The authors attributed this observation to the phenomenon of stealing blood from the short posterior ciliary circulation, which may be a detrimental factor for the chronically ischemic optic nerve caused by glaucoma. However, other authors have presented different results, claiming that, even if such a phenomenon exists, it is not clinically significant [35,40]. Notwithstanding, the effect of the CRA on the macular area seems to be different than that on the optic nerve; blood is not diverted to another area. Importantly, it is not clear whether the CRA still has the features of a choroid vessel, e.g., lacking autoregulation, fast flow and high oxygen concentration. Therefore, it is not known whether it effectively nourishes all layers of the retina.

The state of the retinal microcirculation is affected by both systemic and local factors. Toulouie et al. analysed 594 AMD patients from AREDS and found that AVR was not clearly affected by cilioretinal artery status [41]. In the current study, the parameters of both static and dynamic vascular analysis did not differ depending on the presence of the cilioretinal artery in the AMD and control groups. The diameter of the vessels, as well as their reactivity, is indirectly related to the dysfunction of the vascular endothelium in terms of maintaining the balance between vasoconstrictors and vasodilators. Furthermore, decreased arterial and venous dilatation responses have been linked to various vascular diseases [42]. Importantly, in both of our study groups, there were no differences in the comorbidity of systemic vascular disease or their risk factors between CRA-positive and CRA-negative eyes. There are no previously published studies on the relationship between systemic diseases and CRA prevalence. The cilioretinal artery is a congenital malformation, so intrauterine factors may influence development of the vessel, while cardiovascular diseases are strongly related to lifestyle and environmental factors [43,44]. On the other hand, some human and animal studies seem to suggest that adult vascular disease predisposition may be linked with prenatal factors [45]. The study by Jønsson et al. showed that the CRA was more common among children of mothers who smoked during pregnancy than among children of non-smoking mothers. The authors hypothesized that nicotine and carbon monoxide may be involved in the development of the cilioretinal arteries by inducing fetal retinal hypoxia [46]. In summary, the results of the current study did not show a link between CRA formation and systemic vascular disease predisposition.

An interesting and still unexplained issue is the association of retinal vascular anomalies, such as the CRA with genetic factors. The study by Taarnhøjet et al. analysed a population of 224 healthy twins and concluded that the presence of cilioretinal arteries was predominantly influenced by genetic factors [47]. However, Beneke et al. studied over 800 pairs of twins and estimated that the influence of genetic factors on CRA incidence is much smaller [48]. In a previously published study by our group, we showed that the presence of certain high-risk AMD variants was associated with decreased retinal vascular reactivity, indicating that retinal microcirculation appeared to be associated with genetic background [14]. Here, we found no relationship between the CRA and the presence of high-risk AMD variants. We therefore conclude that AMD and cilioretinal artery formation do not share a common genetic background.

In the current study, for the first time, we evaluated the choroidal parameters of AMD and control eyes in the context of CRA presence. Here, we did not find any significant differences in averaged choroidal parameters in eyes with CRA compared to those without the artery in either the control or AMD group. Similarly, there were no differences in choroidal morphology. To the best of our knowledge, no studies evaluating the effect of the CRA on vascular parameters of the choroid in eyes with AMD have been published thus far. Interestingly, in the control group, the CVI appeared to be higher in the CRA-positive group than in the CRA-negative group. This can be explained by the dilation of existing vessels due to CRA formation. The same relationship was not observed in AMD eyes. Interestingly, Wei et al. showed that, in eyes with degenerative myopia, the presence of the CRA was associated with greater choroidal thickness and better visual acuity [49]. However, it is not known whether the presence of the CRA determines a thicker choroid or vice versa; a thicker choroid is a more abundant source of blood for CRA, which results in a better functional effect. A better supplied choroid (with a higher CVI) may be a more valuable source of blood for CRA and, therefore, for the entire retina.

Interestingly, we observed no significant relationship between the CRA and the presence of pachy-vessels or pachy-choroid features. Similarly, we demonstrated that the incidence of subretinal drusen deposits was not affected by the CRA. A characteristic feature of this subtype of drusen is choroid and retinal thinning, but a causal relationship between choroidal atrophy and SDD has not been conclusively demonstrated. It has been suggested that there is an additional factor that leads to subretinal drusenoid deposit formation and exacerbates choroidal thinning [50]. However, the current study failed to link CRA presence with SDD occurrence. In this study, for the first time, the effect of the cilioretinal artery on various subtypes of drusen, as well as on the presence of pachy-vessels and pachy-choroid features, was assessed.

In a study by Inan et al., the authors did not show statistically significant differences in visual acuity according to the presence of the CRA in either healthy or AMD eyes [51]. This is consistent with the results of our work. In a study by Holm et al., the CRA was found to be related to decreased visual acuity among elderly people aged 67–73 years, especially those with macular pathology. However, the presence of the CRA did not affect the visual function of patients under 63 and over 74 years of age [52].

## 5. Conclusions

In summary, the results of the current study did not show a protective effect of the CRA on AMD incidence and progression. It was shown that the cilioretinal artery may affect the severity of AMD; however, the mechanism of this phenomenon is unclear. We did not find any relationship between CRA presence and choroidal and retinal averaged parameters and morphology in either AMD or healthy control eyes. In addition, high-risk AMD variants were not associated with the occurrence of the CRA.

## Figures and Tables

**Table 1:** Characteristics of the patients in the study groups. Bold values indicate significance at *p* < 0.05.

Parameter	AMD Group			Control Group		
	CRA Present	CRA Absent	*p*	CRA Present	CRA Absent	*p*
Number of subjects	67	220	0.313	27	83	0.27
Sex (male/female)	16.8/27.2	83.2/72.8	**0.05**	28.6/23.2	71.4/76.8	0.61
Age (years) (mean ± SD)	72.39 (7.89)	73.31 (8.11)	0.31	73.96 (6.57)	75.52 (5.28)	0.27
Hypertension [%]	71.88	62.38	0.18	66.67	74.24	0.6
Duration of hypertension (years) (mean ± SD)	9.91 (9.48)	8.15 (9.89)	0.09	9.96 (12.78)	8.99 (8.57)	0.82
History of ischemic heart disease (%)	10.94	16.92	0.33	16.67	9.09	0.45
Duration of ischemic heart disease (years) (mean ± SD)	0.75 (2.92)	1.4 (4.55)	0.51	1.46 (5.0)	0.58 (2.52)	0.66
History of myocardial infarction (%)	1.56	7.96	0.08	8.33	6.06	0.66
Stroke (%)	4.69	2	0.37	4.17	3.03	1.0
Peripheral artery disease [%]	1.56	6.47	0.2	12.55	4.5	0.34
Current smokers [%]	12.5	16.34	0.55	4.17	6.15	1.0
Former smokers [%]	42.19	55.94	0.06	20.83	34.85	0.3
Period without smoking [years] (mean ± SD)	4.47 (8.79)	7.01 (15)	0.1	2.33 (5.42)	6.49 (11.3)	0.16
Smoking pack-years (mean ± SD)	12.74 (21.22)	14.45 (18.76)	0.13	2.08 (4.82)	7.55 (15.17)	0.13
BMI [kg/m^2^] (mean ± SD)	27.8 (4.66)	26.68 (3.97)	0.08	26.71 (2.92)	26.48 (4.02)	0.63
WHR [arbitrary units] (mean ± SD)	0.9 (0.09)	0.89 (0.09)	0.41	0.88 (0.1)	0.88 (0.09)	0.94
MAP [mmHg] (mean ± SD)	97.96 (11.61)	97.28 (11.0)	0.95	99.93 (9.37)	97.96 (9.57)	0.38
Physical activity [MET] (mean ± SD)	1946.28 (2883.21)	1513.57 (1871.18)	0.61	1489.19 (1497.22)	1386.21 (1566.13)	0.75

Mann–Whitney U test/Fisher’s exact test.

**Table 2:** Phenotypic characteristics of AMD eyes with and without cilioretinal arteries. Statistically significant results are marked in bold.

Clinical Parameter		CRA Present	CRA Absent	*p*-Value
Pachychoroid (Y/N)		6/72	28/492	0.577
Pachyvessels (Y/N)		22/56	129/392	0.608
Pachydrusen (Y/N)		5/73	31/492	0.93
Subretinal drusenoid deposits (SDD) (Y/N)		24/54	142/379	0.609
Soft drusen (Y/N)		32/31	249/161	0.168
Drusen size (µm) [mean ± SD]		2.08 ± 0.96	1.88 ± 1.14	0.293
AMD stage	Early (yes [%])	47.37	21.38	**0.016**
	Intermediate (yes [%])	47.37	56.60	
	Late (yes [%])	5.26	22.01	
Geographic atrophy (Y/N)		1/22	35/183	0.216
CNV (Y/N)		16/6	129/55	1.000

Mann–Whitney/chi-squared or Fisher’s exact test.

**Table 3:** Differences between clinical parameters of eyes in the AMD and control groups according to the presence of the cilioretinal artery (CRA). BCDVA—best-corrected distance visual acuity; AV—average volume; ATC—average thickness center; AVC—average volume center, CVI—choroidal vascularity index, AVR—arteriovenous ratio, DAA—dynamic analysis of arteries, DAV—dynamic analysis of veins. The values are presented as the median (IQR). Statistically significant results are marked in bold.

Clinical Parameter	AMD Group			Control Group		
	CRA Present (IQR)	CRA Absent (IQR)	*p*	CRA Present (IQR)	CRA Absent (IQR)	*p*
BCDVA (logMAR)	0.3 (0.6)	0.4 (0.5)	0.3	0.2 (0.3)	0.2 (0.3)	0.72
AV (mm^3^)	7.11 (3.42)	6.71 (3.23)	0.09	7.29 (2.03)	6.83 (2.93)	0.63
ATC (μm)	311 (146)	273 (142)	0.14	277 (111)	269.5 (118)	1.0
AVC (mm^3^)	0.24 (0.11)	0.21 (0.11)	0.14	0.22 (0.08)	0.21(0.09)	1.0
CVI	0.65 (0.03)	0.65 (0.04)	0.92	0.67 (0.04)	0.66 (0.03)	**0.04**
CRT (μm)	286.5 (61)	289 (84)	0.65	265 (28)	276 (27)	0.23
AVR	0.86 (0.1)	0.85 (0.09)	0.28	0.86 (0.06)	0.85 (0.09)	0.91
DAA (%)	2.85 (3.45)	2.8 (3.1)	0.87	3.1(2.7)	3 (3.1)	0.98
DAV (%)	3.9 (3)	4.1 (2.7)	0.73	4.1 (2)	4 (3.2)	0.93

Mann–Whitney/Fisher’s exact test.

**Table 4:** Distribution of cilioretinal artery (CRA) presence in at least one eye among patients with different high-risk AMD genotypes.

Tested SNP	Genotype	% of AMD Patients with CRA	% of AMD Patients without CRA	*p*-Value	% of Controls with CRA	% of Controls without CRA	*p*-Value
*CFH* Y402H	TT	20.75%	79.25%	0.38	23.68%	76.32%	0.46
	TC	25.71%	74.29%		32.56%	67.44%	
	CC	17.44%	82.56%		16.67%	83.33%	
*ARMS2* A69S	GG	24.69%	75.31%	0.69	27.78%	72.22%	0.57
	GT	19.69%	80.31%		27.78%	72.22%	
	TT	21.62%	78.38%		0.00%	100.00%	

Chi-squared test.

## Data Availability

The data that support the findings of this study are available on request from the corresponding author, A.M.
